# Emergence and persistent spread of carbapenemase-producing *Klebsiella pneumoniae* high-risk clones in Greek hospitals, 2013 to 2022

**DOI:** 10.2807/1560-7917.ES.2023.28.47.2300571

**Published:** 2023-11-23

**Authors:** Kyriaki Tryfinopoulou, Marius Linkevicius, Olga Pappa, Erik Alm, Kleon Karadimas, Olov Svartström, Michalis Polemis, Kassiani Mellou, Antonis Maragkos, Alma Brolund, Inga Fröding, Sophia David, Alkiviadis Vatopoulos, Daniel Palm, Dominique L Monnet, Theoklis Zaoutis, Anke Kohlenberg, Greek CCRE study group, Members of the Greek CCRE study group, Efstathia Perivolioti, Petros Andrikogiannopoulos, Athina Argyropoulou, Spyros Pournaras, Sofia Vourli, Panagiota Christina Georgiou, Konstantinos Stamoulos, Gerasimos-Socrates Christodoulatos, Iosif Ntarouis, Sofia Varveraki, Christos Konsolakis, Kalliopi Panteli, Olympia Zarkotou, Nektaria Rekleiti, Elisavet Kousouli, Marina Papadogianni, Kalina Zervaki, Tatiana Gavrilidi, Maria Panopoulou, Nikolaos Lemonakis, Anastasia Grapsa, Theodoros Karampatakis, Eleni Kandilioti, Tania Gkiti, Paraskevi Karapavlidou, Christos Sideris, Evangelia Dafa, Konstantina Gartzonika, Christos Kittas, Nikoletta Karavasili, Stelios Xytsas, Katerina Tsilipounidaki, Efthymia Petinaki, Aggeliki Pasxali, Glykeria Sorovou, Efrosini Krikoni, Christina Bartzavali, Fevronia Kolonitsiou, Fotini Paliogianni, Christina Kaminioti, Christina Mparka, Dimitra Petropoulou, Panagiota Giannopoulou, Nikoletta Charalampaki, Penelope Kappou

**Affiliations:** 1 National Public Health Organization, Athens, Greece; 2 European Centre for Disease Prevention and Control, Stockholm, Sweden; 3 Public Health Agency of Sweden, Stockholm, Sweden; 4 Centre for Genomic Pathogen Surveillance, Pandemic Sciences Institute, University of Oxford, Oxford, United Kingdom; 5 School of Public Health, University of West Attica, Athens, Greece; 6 The Members of the Greek CCRE study group are listed under Collaborators; * These authors contributed equally to this work and share first authorship

**Keywords:** carbapenem-resistant Enterobacterales, carbapenemase, KPC, NDM, surveillance, whole genome sequencing

## Abstract

**Background:**

Preliminary unpublished results of the survey of carbapenem- and/or colistin-resistant Enterobacterales (CCRE survey) showed the expansion of carbapenemase-producing *Klebsiella pneumoniae* (CPKP) sequence type (ST) 39 in 12 of 15 participating Greek hospitals in 2019.

**Aim:**

We conducted a rapid survey to determine the extent of spread of CPKP high-risk clones in Greek hospitals in 2022 and compare the distribution of circulating CPKP clones in these hospitals since 2013.

**Methods:**

We analysed whole genome sequences and epidemiological data of 310 *K. pneumoniae* isolates that were carbapenem-resistant or ‘susceptible, increased exposure’ from Greek hospitals that participated in the European survey of carbapenemase-producing *Enterobacteriaceae* (EuSCAPE, 2013–2014), in the CCRE survey (2019) and in a national follow-up survey (2022) including, for the latter, an estimation of transmission events.

**Results:**

Five *K. pneumoniae* STs including ST258/512 (n = 101 isolates), ST11 (n = 93), ST39 (n = 56), ST147 (n = 21) and ST323 (n = 13) accounted for more than 90% of CPKP isolates in the dataset. While ST11, ST147 and ST258/512 have been detected in participating hospitals since 2013 and 2014, KPC-2-producing ST39 and ST323 emerged in 2019 and 2022, respectively. Based on the defined genetic relatedness cut-off, 44 within-hospital transmission events were identified in the 2022 survey dataset, with 12 of 15 participating hospitals having at least one within-hospital transmission event.

**Conclusion:**

The recent emergence and rapid spread of new high-risk *K. pneumoniae* clones in the Greek healthcare system related to within-hospital transmission is of concern and highlights the need for molecular surveillance and enhanced infection prevention and control measures.

**Key public health message**

**What did you want to address in this study and why?**

A new highly drug-resistant clone of carbapenemase-producing *Klebsiella pneumoniae* had been detected in 12 of 15 Greek hospitals participating in a European genomic surveillance project in 2019. We looked at a collection of 10 carbapenem-resistant *K. pneumoniae* isolates per hospital to see how widely these bacteria had spread in 2022; such timely information could be used for infection prevention and control.

**What have we learnt from this study?**

The carbapenemase-producing *K. pneumoniae* detected in Greek hospitals over a 10-year period mainly belonged to a small number of so-called ‘high-risk clones’ known for their antimicrobial resistance and capacity to spread in healthcare settings. We also noted that new high-risk clones have recently emerged and spread rapidly throughout hospitals in the country.

**What are the implications of your findings for public health?**

Infection prevention and control measures need to be enhanced immediately to prevent further spread of highly-drug resistant *K. pneumoniae* and similar resistant bacteria in hospitals. This study is a model for rapid national molecular surveillance studies that could also be applied in other countries and settings.

## Introduction

*Klebsiella pneumoniae* is a frequent cause of healthcare-associated infections including pneumonia, urinary tract, wound and bloodstream infections [1]. Carbapenem-resistant *K. pneumoniae* infections are associated with a high mortality [2], primarily due to delayed administration of effective treatment and limited treatment options. Carbapenem resistance in Enterobacterales including *K. pneumoniae* has been recognised as a notable concern for patients and healthcare systems in countries of the European Union/European Economic Area (EU/EEA) [3]. In addition, the transmission of high-risk clones of carbapenem-resistant *K. pneumoniae* in healthcare settings has been identified as the major cause of the spread in EU/EEA countries [4].

National whole-genome sequencing (WGS)-based molecular surveillance for the detection and control of these clones is at various stages of implementation [5]. European-level genomic surveillance of carbapenem-resistant *K. pneumoniae* high-risk clones has so far been based on structured multi-country surveys with central WGS and analysis. The first such survey was the European survey of carbapenemase-producing *Enterobacteriaceae* (EuSCAPE) which collected isolates and data in 36 countries in 2013 and 2014 [4,6]. It was followed by the survey of carbapenem-resistant and/or colistin-resistant Enterobacterales (CCRE survey) which collected isolates and data in 36 countries in 2019 [7].

Preliminary unpublished results of the CCRE survey showed the expansion of a new clade of carbapenemase-producing *K. pneumoniae* (CPKP) sequence type (ST) 39 carrying *bla*_KPC-2_ and/or *bla*_VIM-1_ in participating Greek hospitals. This clade was not present among isolates collected in 10 Greek hospitals in EuSCAPE in 2013 and 2014 [4] but was detected in 12 of 15 Greek hospitals participating in the CCRE survey in 2019 [7]. The CCRE survey results became available only after the COVID-19 pandemic in 2022. At this time, the extent of the spread of *K. pneumoniae* ST39 was not known and required further investigation to generate more timely data for infection prevention and control (IPC). A national molecular follow-up survey was therefore conducted in 2022. Here we report the results of this follow-up survey which, combined with EuSCAPE and the CCRE survey data for Greece, allows tracking of high-risk clones in Greek hospitals over a 10-year period.

## Methods

### Data and isolate collection

The detailed methods and definitions for the EuSCAPE and the CCRE survey are outlined in EuSCAPE publications and epidemiological and microbiological protocols for the CCRE survey [6-9]. Of note, both surveys aimed to achieve geographical representativeness, EuSCAPE by recruiting a defined number of hospitals depending on the country’s population and the CCRE survey by including one hospital per Nomenclature of Territorial Units for Statistics (NUTS)-level-2 region. Both collected up to 10 consecutive carbapenem-resistant (R) or carbapenem-‘susceptible, increased exposure’ (I) *K. pneumoniae* or *E. coli* isolates from individual patients per hospital as well as up to 10 carbapenem-‘susceptible, standard dosing regimen’ (S) comparator isolates of the same species.

From July to September 2022, we used a modified CCRE survey protocol to conduct a molecular follow-up survey in 15 Greek hospitals that had participated in the CCRE survey [7]. In short, the Greek National Reference Laboratory invited the hospitals to submit up to 10 consecutive *K. pneumoniae* isolates resistant to any carbapenem (based on European Committee on Antimicrobial Susceptibility Testing clinical breakpoints [10]) from individual patients accompanied by epidemiological and microbiological data. Phenotypic antimicrobial susceptibility testing (AST) of isolates was conducted at hospital microbiology laboratories with minimum inhibitory concentration (MIC) determination by VITEK 2 or disk diffusion for all analysed antibiotics except for colistin, which was evaluated by the commercial broth microdilution test available for routine use in each hospital. The epidemiological variables collected for this follow-up survey were the date of sampling, the healthcare institution submitting the sample, the sample type, the clinical relevance (infection or carriage), the status of the patient (inpatient or outpatient), the type of acquisition (community- or hospital-acquired), and age and sex of the patient. 

Results from WGS and AST and epidemiological data of these isolates were combined with data on carbapenem-resistant (R) or carbapenem-‘susceptible, increased exposure’ (I) from Greek hospitals in the EuSCAPE and CCRE survey datasets.

### Whole genome sequencing data analysis

Sequencing reads were assembled using SPAdes v3.15.3 [11] and analysed in Pathogenwatch [12]. After quality control, 310 carbapenem-R/I isolates were included in this study: 40 from EuSCAPE (restricted to hospitals that also participated in the CCRE and follow-up surveys), 128 from the CCRE survey, and 142 from the follow-up survey. A neighbour-joining phylogenetic tree based on core genome single nucleotide polymorphisms (cgSNPs) was constructed using Pathogenwatch [12]. In addition, STs (Institute Pasteur scheme) [13] and antibiotic resistance genes were determined using Kleborate v2.3.0 [14].

To estimate recent within-hospital transmission, we used the 2022 follow-up survey isolate pairs with ≤ 80 cgSNP pairwise distances determined in Pathogenwatch [12] within the same *K. pneumoniae* ST. Each pair was annotated as ‘same hospital’ or ‘different hospitals’ and cgSNP distances were calculated. We generated histograms showing distances for ‘same hospital’ and ‘different hospitals’ pairs. Transitions in the slope of the cumulative proportions per distance were used as signs of transition between phenomena and cluster cut-off candidates.

## Results

### Participation and geographical representativeness

All 15 hospitals invited to participate in the follow-up survey sent isolates accompanied with epidemiological data. The number of isolates included in the final dataset ranged from five to 14 isolates per hospital with sampling dates between 22 February and 3 October 2022 (Table). The 15 hospitals participating in the CCRE follow-up survey were located throughout the country, with 10 of 13 Greek NUTS-2 units represented with at least one hospital. For nine of these NUTS-2 units (EL43, EL51, EL52, EL53, EL54, EL61, EL62, EL63 and EL65), one hospital each was included, whereas for Attica (EL30), six hospitals were included taking into account that almost half of the Greek population lives in the main metropolitan region of Attica and also that this region receives many referrals for tertiary care from throughout the country. Combining all three surveys, this resulted in 128 carbapenem-R/I *K. pneumoniae* isolates in the dataset originating from EL30, while the other nine NUTS-2 units were covered with 13–30 isolates each (Table).

**Table:** Carbapenem-R/I *Klebsiella pneumoniae* isolates from 15 hospitals participating in two ECDC surveys and a national follow-up survey, Greece, 2013–2022 (n = 310)

NUTS-2 unit	Hospital identifier^a^	Number of included carbapenem-R/I *K. pneumoniae* isolates			
		EuSCAPE (2013–2014)	CCRE survey (2019)	Follow-up survey (2022)	Total
EL30	GR01	NP	9	9	18
	GR02	NP	10	9	19
	GR03	9	9	10	28
	GR04	6	9	10	25
	GR05	NP	9	8	17
	GR18	5	6	10	21
EL43	GR07	5	10	10	25
EL51	GR08	NP	9	5	14
EL52	GR09	6	9	10	25
EL53	GR11	NP	3	10	13
EL54	GR12	NP	8	10	18
EL61	GR13	NP	10	9	19
EL62	GR14	9	7	14^b^	30
EL63	GR15	NP	10	10	20
EL65	GR17	NP	10	8	18
**Total**	**15**	**40**	**128**	**142**	**310**

CCRE: carbapenem- and/or colistin-resistant Enterobacterales; ECDC: European Centre for Disease Prevention and Control; EuSCAPE: European survey of carbapenemase-producing *Enterobacteriaceae*; NP: not participating; NUTS-2: Nomenclature of territorial units for statistics 2; I: susceptible, increased exposure; R: resistant.

^a^ The hospitals were selected based on their participation in the CCRE survey: 18 Greek hospitals had been invited to participate but only 15 responded and sent isolates. The three hospitals not responding to that survey, namely GR06, GR10, and GR16, are therefore not included in the dataset.

^b^ For this hospital, 14 isolates were included, deviating from the protocol requesting only up to 10 isolates per hospital.

### Distribution of sequence types

**Figure 1:**
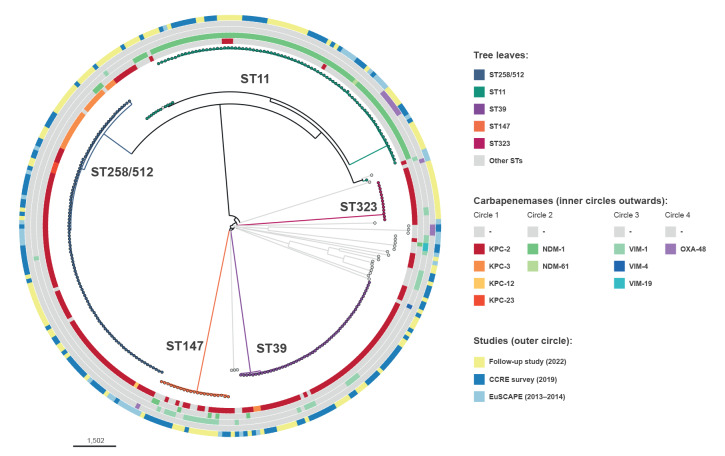
Core-genome single nucleotide polymorphism-based phylogenetic tree of carbapenem-R/I *Klebsiella pneumoniae* isolates collected in 15 hospitals participating in two ECDC surveys and a national follow-up survey, Greece, 2013–2022 (n = 310)

CCRE: carbapenem- and/or colistin-resistant Enterobacterales; ECDC: European Centre for Disease Prevention and Control; EuSCAPE: European survey of carbapenemase-producing *Enterobacteriaceae*; ST: sequence type.

**Figure 2:**
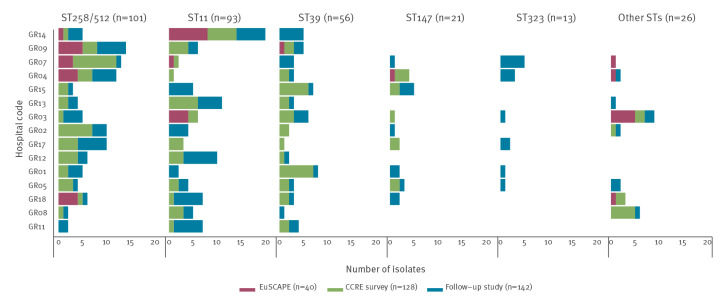
Distribution of the five most frequent sequence types of carbapenem-R/I *Klebsiella pneumoniae* isolates collected in 15 hospitals participating in two ECDC surveys and a national follow-up survey, Greece, 2013–2022 (n = 310)

CCRE: carbapenem- and/or colistin-resistant Enterobacterales; ECDC: European Centre for Disease Prevention and Control; EuSCAPE: European survey of carbapenemase-producing *Enterobacteriaceae*; ST: sequence type.

**Figure 3:**
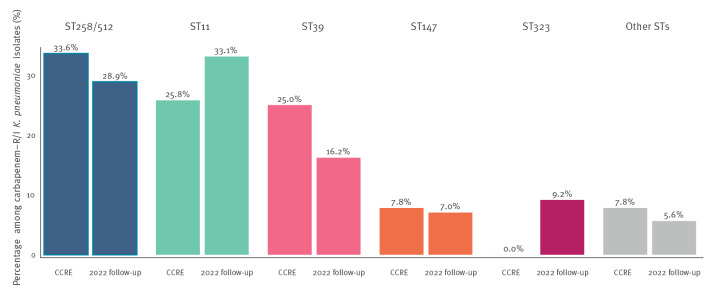
Distribution of the five most frequent sequence types of carbapenem-R/I *Klebsiella pneumoniae* isolates collected in 15 hospitals participating in the CCRE survey and a national follow-up survey, Greece, 2013–2022 (n = 270)

CCRE: carbapenem- and/or colistin-resistant Enterobacterales; ST: sequence type.

Combining data of the three surveys showed that, in Greece, CPKP mainly belonged to five STs which represented more than 90% (n = 284) of carbapenem-R/I isolates in the dataset. The most frequent ST of carbapenem-R/I *K. pneumoniae* was ST258/512 (n = 101 isolates) followed by ST11 (n = 93), ST39 (n = 56), ST147 (n = 21) and ST323 (n = 13), while all other STs were present with less than 10 isolates in the dataset (Figure 1). The three most frequent *K. pneumoniae* STs, i.e. ST258/512, ST11 and ST39, were detected in all participating hospitals, while the hospital distribution of *K. pneumoniae* ST147, ST323 and all other STs was more variable (Figure 2). The distribution of STs changed over time (Figure 3). Within clonal complex (CC)258, *K. pneumoniae* ST258/512 was replaced by ST11 as the most frequent ST in 2022. Moreover, *K. pneumoniae* ST39 and ST323 carrying *bla*_KPC-2_ were absent in 2013 and 2014 but emerged, respectively, in 2019 and 2022 (Figures 1 and 3).

*bla*_NDM-61_ is a novel NDM gene allele detected in this study*.*

ST258/512 includes three isolates with single-locus variants of ST258.

ST258/512 includes three isolates with single-locus variants of ST258.

### Characteristics of *Klebsiella pneumoniae* ST39

All 56 ST39 isolates with predicted carbapenem resistance carried one single or several carbapenemase genes, including *bla*_KPC-2_ (alone or in combination with *bla*_VIM-1_ or *bla*_NDM-1_), *bla*_KPC-3_, *bla*_VIM-1_ or *bla*_VIM-4_, as well as mutations of genes encoding for major outer membrane porins, i.e. OmpK35 (amino acid sequence truncation) and OmpK36 (Gly115-Asp116 insertion). In addition, these isolates were also predicted to be fluoroquinolone-resistant since chromosomal mutations in the genes encoding for GyrA (S83I and D87N) and for ParC (S80I) were detected. Of these, 47 isolates were predicted to be colistin-resistant due to truncation in the MgrB regulator alone (n = 46) or in combination with truncation in PmrB (n = 1). Of these 47 isolates, 46 were harbouring genes for aminoglycoside-modifying enzymes among which more than half were *aac(6’)-Ib*, *aadA2*, *aac(3)-IId*, *aadA* and/or *aph(3)-Ia*. For predicted virulence, most ST39 isolates (n = 46) carried the siderophore yersiniabactin gene *ybt14* mobilised by the *K. pneumoniae* integrative conjugative element ICE*Kp5*.

### Distribution of carbapenemase genes and phenotypic antimicrobial susceptibility testing results

**Figure 4:**
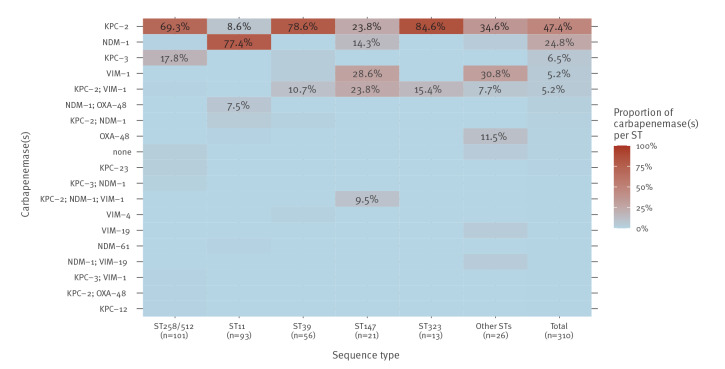
Distribution of carbapenemases by sequence type of carbapenem-R/I *Klebsiella pneumoniae* isolates collected in 15 hospitals participating in two ECDC surveys and a national follow-up survey, Greece, 2013–2022 (n = 310)

CCRE: carbapenem- and/or colistin-resistant Enterobacterales; ECDC: European Centre for Disease Prevention and Control; EuSCAPE: European survey of carbapenemase-producing *Enterobacteriaceae*; ST: sequence type.

The most frequent carbapenemase gene, detected in 171 (55.2%) carbapenem-R/I isolates, was *bla*_KPC-2_. In 147 isolates, it was the only detected carbapenemase gene. In the remaining 24 isolates, *bla*_KPC-2_ was present in various combinations with *bla*_NDM-1_, *bla*_OXA-48_ and/or *bla*_VIM-1_. The distribution of carbapenemase genes was associated with specific STs. In our dataset, the *K. pneumoniae* STs with the highest proportions of isolates only carrying *bla*_KPC-2_ were ST323 (n = 11/13), ST39 (n = 44/56), and ST258/512 (n = 70/101) (Figure 4). The second most frequent carbapenemase gene in carbapenem-R/I isolates was *bla*_NDM-1_ detected in 94 (30.3%) isolates, of which 77 carried only *bla*_NDM-1_ and 17 showed various combinations with other carbapenemase genes. The *K. pneumoniae* ST with the highest proportion (n = 72, 77.4%) of isolates carrying only *bla*_NDM-1_ was ST11 (Figure 4). A new NDM gene variant, namely *bla*_NDM-61_, was detected in one ST11 isolate from the 2022 follow-up survey.

All of the included 310 isolates were in the category R or I for at least one carbapenem as per inclusion criteria. Among these, 295 of 300 tested isolates (98.3%) were resistant to ciprofloxacin and 278 of 278 (100%) resistant to at least one aminoglycoside. Overall, this resulted in 272 of 278 isolates (97.8%) with complete AST results being confirmed as multidrug-resistant [15]. In addition, 80 of 214 (37.4%) tested isolates were resistant to colistin. Results for ceftazidime/avibactam were available only for the CCRE survey and the 2022 follow-up survey, and 99 of 227 (43.6%) tested isolates were resistant, of which 81 carried a metallo-β-lactamase gene.

### Epidemiological results

Of 247 carbapenem-R/I *K. pneumoniae* isolates with available information on infection or carriage, 173 (70.0%) were associated with infection, 61 (24.7%) with carriage, and 13 (5.3%) were of undetermined clinical significance. For the isolates associated with infection, the most frequently reported sampling sites were blood (n = 65, 37.6%), urine (n = 63, 36.4%), respiratory tract (n = 18, 10.4%) and wound (n = 16, 9.2%), while 11 (6.4%) isolates originated from various other body sites. The majority of isolates associated with infection originated from patients ≥ 60 years of age (n = 136, 78.6%) and from male patients (n = 104, 60.1%). Of 216 isolates with available information on the type of acquisition, 165 (76.4%) were classified as hospital-acquired and 51 (23.6%) as community-acquired.

### Within-hospital transmission

**Figure 5:**
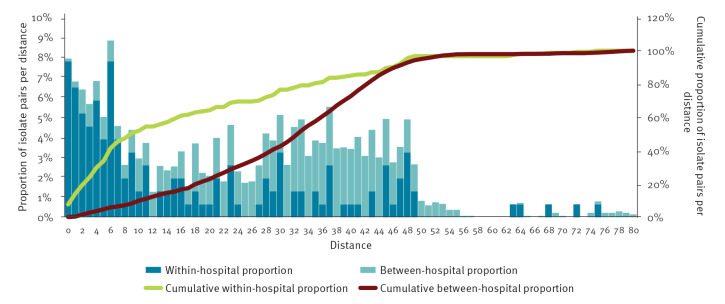
Comparison of ‘same hospital’/’different hospitals’ isolate pairs using Pathogenwatch core genome single-nucleotide polymorphisms (cgSNPs)

The optimum cut-off for ‘same hospital’/’different hospitals’ isolate pairs was determined to be between 7 and 11 cgSNP (Figure 5). To use a more conservative estimate, the cut-off for recent within-hospital transmission events was set to ≤ 8 cgSNPs and resulting pairs were evaluated for the most likely transmission pathway. Using this cut-off, 44 within-hospital transmission events were identified in the 2022 follow-up survey dataset, with 12 of 15 participating hospitals having at least one within-hospital transmission event. The number of within-hospital transmission events per hospital ranged from one to six and included 12 clusters of more than one related transmission event. Of these clusters, six were associated with ST11, two with ST258/512, two with ST39, one with ST323 and one with ST147. All within-hospital transmission events involved *K. pneumoniae* high-risk clones, most frequently ST11 (n = 22 transmission events), followed by ST258/512 and ST39 (n = 7 each), ST323 (n = 6) and ST147 (n = 2).

## Discussion

The worldwide spread of CPKP is driven by the transmission of international high-risk clones in healthcare facilities [4,16]. This study documents the dissemination of high-risk clones of CPKP in the participating Greek hospitals over a 10-year period and shows the persistent spread of previously established as well as newly emerging high-risk clones. Three of the frequent *K. pneumoniae* STs in this dataset, ST258/512, ST11 and ST147, are known international high-risk clones also dominating in other EU/EEA countries and worldwide [4,16,17]. *Klebsiella pneumoniae* ST258/512 and ST11 were widely distributed in all participating Greek hospitals and persistently detected over 10 years. While *K. pneumoniae* ST258/512 was the most frequent ST among CPKP isolates in Greece in 2009 and 2010 [18] and dominated in ECDC surveys in 2013 and 2014 and in 2019, *K. pneumoniae* ST11 was the most frequent in 2022. It is so far unclear whether CPKP ST11 has a competitive advantage over ST258/512 and if this trend will continue. Nevertheless, the large number of within-hospital transmission events associated with ST11 suggests that, once introduced in a hospital, this clone has a high potential for spread. An advantage of ST11 may also be its frequent association with *bla*_NDM-1_ which confers resistance to ceftazidime/avibactam in a context of increasing use of this combination antibiotic [19].

In Greece, highly drug-resistant *K. pneumoniae* ST39 carrying *bla*_KPC-2_ and *bla*_VIM-1_, were recently reported in isolates from 2018 and 2019 [20]. Our study shows that *K. pneumoniae* ST39 carrying *bla*_KPC-2_ has already spread to all 15 participating Greek hospitals within a short period and can therefore be considered a high-risk clone. This clone has so far only rarely been described outside of Greece; however, unpublished data from the CCRE survey show five sporadic isolates in five other EU/EEA countries and further investigation of potential cross-border spread is ongoing. Import of *K. pneumoniae* ST39 carrying *bla*_KPC-2_ from Greece to Finland has also been described [21]. Of note, development of resistance to ceftazidime/avibactam during treatment has previously been reported for this clone [21,22]. Even in a country with long-standing endemicity for CPKP such as Greece, the emergence of new high-risk clones is relevant as this is the starting point for further spread of these clones which usually have additional antimicrobial resistance mechanisms and/or are better adapted to transmission in healthcare settings. Our results document the steps in the emergence and spread of CPKP ST39 as a high-risk clone, through acquisition of resistance genes (mainly *bla*_KPC-2_ but also *bla*_VIM-1_) and mutations of genes encoding outer membrane porins (i.e. OmpK35/OmpK36) in a setting with high antibiotic pressure, and within-hospital and between-hospitals transmission events in a national healthcare system.

Finally, another *K. pneumoniae* ST harbouring *bla*_KPC-2_, namely ST323, that was not detected in Greece in 2019, appears to have rapidly spread to six Greek hospitals in 2022, potentially repeating the rapid increase of *K. pneumoniae* ST39. However, the spread of ST323 has mainly involved hospitals in Attica, Peloponnese and Crete, but not yet the six participating hospitals in northern and central Greece. Estimation of within-hospital transmission shows that the persistent spread of previously established CPKP STs and the rapid spread of new CPKP STs can probably be attributed to frequent transmission events in hospitals. In addition, the high percentage of CPKP isolates associated with infections in this study highlights the relevance of CPKP spread for clinical care. Phenotypic AST results show high levels of multidrug resistance in CPKP isolates, reducing available treatment options. However, it is a limitation of this study that these isolates have not been tested for newer antimicrobial substances, e.g. meropenem/vaborbactam, imipenem/relebactam, and cefiderocol.

Whole genome sequencing is a major development, which contributes to surveillance and control of antimicrobial resistance through early detection of transmission events and outbreaks, tracking of transmission pathways and the characterisation of involved pathogens and resistance mechanisms [23,24]. The EuSCAPE and the CCRE survey had been designed mainly with the aim of tracking high-risk clones and genetic resistance elements of public health concern on the European level and not for national surveillance or investigation of hospital outbreaks. Because only one hospital per NUTS-2 unit was included and the number of consecutive carbapenem-R/I *K. pneumoniae* isolates was restricted to 10 per hospital, these surveys had a limited geographical resolution and a limited power to investigate outbreaks in participating hospitals. However, in the absence of comprehensive WGS-based surveillance, data from the two surveys can provide valuable insights into the national epidemiology of CPKP in EU/EEA countries and allow the detection of newly emerging resistance threats as shown in this study for Greece.

The limitation of the EuSCAPE and the CCRE survey from an IPC perspective has so far been the delay between isolate collection and availability of WGS results, which is too long for an immediate impact on CPKP control in participating hospitals and countries. The 2022 follow-up survey in Greece showed, however, that a modified protocol can be implemented much faster at national level and generate near real-time data useful for IPC purposes. Still, the information about outbreaks and high-risk areas as well as populations remains very limited. Therefore, the overarching goal continues to be the implementation of comprehensive WGS-based surveillance targeting multidrug-resistant organisms in EU/EEA countries. While establishment of the required capacity is ongoing in many countries, this 2022 follow-up survey in Greece could serve as a model for rapid, targeted studies of the WGS-based epidemiology not only of CPKP, but potentially other multidrug-resistant organisms of concern, in healthcare settings in one or several EU/EEA countries. Identified new or increasing resistance threats, potential outbreaks and high-risk areas for transmission could then be followed up with more targeted and detailed epidemiological and genomic studies to determine the sources and transmission pathways.

Greece has a national surveillance for healthcare-associated infections caused by multidrug-resistant Gram-negative organisms in public and private hospitals called PROKROUSTIS, mandatory since 2014. As part of this system, national IPC guidelines have been provided and continuously updated, including guidelines for patient isolation. While most hospitals also perform screening for Gram-negative multidrug-resistant organisms at admission to intensive care, reporting is not mandatory and detailed information on screening practices and related results is therefore not available at national level.

To enhance IPC in Greek hospitals, a variety of additional actions could be considered, including (i) strengthening of IPC committees in hospitals with administrative support, (ii) increasing the number of IPC nurses at national level, (iii) establishing training modules for IPC specialists at medical schools and enhancing training for all healthcare professionals on IPC and antimicrobial stewardship (AMS), (iv) implementing new protocols for prevention of central line-associated bloodstream infections and catheter-associated urinary tract infections and (v) upgrading the surveillance system with a national database capable of mining information directly from hospital and laboratory information systems. In addition, the mandatory electronic prescription system for medical doctors at primary care level and establishment of AMS committees at hospital level would improve antibiotic usage.

## Conclusion

The recent emergence and spread of *K. pneumoniae* ST39 and ST323 carrying predominantly *bla*_KPC-2_ in Greece is not only relevant because of their highly drug-resistant profile, but also as an example of rapid spread of newly emerging antimicrobial resistance threats throughout a hospital network. Similarly, rapid interhospital and interregional spread has been documented for CPKP and other emerging antimicrobial resistance threats in other EU/EEA countries [25-27]. This situation is of concern and highlights the need for molecular surveillance and enhanced IPC measures in hospitals in Greece and in other EU/EEA countries, and more generally increased efforts to control antimicrobial resistance in the EU/EEA and beyond.

## Collaborators (Members of the Greek CCRE study group):

Efstathia Perivolioti, Petros Andrikogiannopoulos, Athina Argyropoulou (Evaggelismos General Hospital of Athens, Attiki), Spyros Pournaras, Sofia Vourli, Panagiota Christina Georgiou (Attikon University Hospital of Athens, Attiki), Konstantinos Stamoulos, Gerasimos-Socrates Christodoulatos, Iosif Ntarouis (Sismanogleio General Hospital of Athens, Attiki), Sofia Varveraki, Christos Konsolakis, Kalliopi Panteli (Asklepieio General Hospital of Voula, Attiki), Olympia Zarkotou, Nektaria Rekleiti, Elisavet Kousouli (Tzaneio General Hospital of Piraeus, Attiki), Marina Papadogianni, Kalina Zervaki, Tatiana Gavrilidi (General Hospital of Chania, Crete), Maria Panopoulou, Nikolaos Lemonakis, Anastasia Grapsa (University Hospital of Alexandroupolis, Thrace), Theodoros Karampatakis, Eleni Kandilioti, Tania Gkiti (Papanikolaou General Hospital of Thessaloniki, Central Macedonia), Paraskevi Karapavlidou, Christos Sideris, Evangelia Dafa (General Hospital of Kastoria, Western Macedonia), Konstantina Gartzonika, Christos Kittas, Nikoletta Karavasili (University Hospital of Ioannina, Epirus), Stelios Xytsas, Katerina Tsilipounidaki, Efthymia Petinaki (University Hospital of Larissa, Thessaly), Aggeliki Pasxali, Glykeria Sorovou, Efrosini Krikoni (General Hospital of Corfu, Ionian islands), Christina Bartzavali, Fevronia Kolonitsiou, Fotini Paliogianni (University Hospital of Patras, Western Greece), Christina Kaminioti, Christina Mparka, Dimitra Petropoulou (General Hospital of Tripoli, Peloponnese), Panagiota Giannopoulou, Nikoletta Charalampaki, Penelope Kappou (Thriassio General Hospital of Attiki).
